# Thromboelastography and Thromboelastometry in Assessment of Fibrinogen Deficiency and Prediction for Transfusion Requirement: A Descriptive Review

**DOI:** 10.1155/2018/7020539

**Published:** 2018-11-25

**Authors:** Henry T. Peng, Bartolomeu Nascimento, Andrew Beckett

**Affiliations:** ^1^Defence Research and Development Canada, Toronto Research Centre, Toronto, Ontario, Canada; ^2^Research Institute of Xi'an Jiaotong University, Hangzhou, Zhejiang, China; ^3^Sunnybrook Health Sciences Centre, Department of Surgery, University of Toronto, Toronto, Canada; ^4^Royal Canadian Medical Services, Ottawa, Ontario, and McGill University, Montreal, Quebec, Canada

## Abstract

Fibrinogen is crucial for the formation of blood clot and clinical outcomes in major bleeding. Both Thromboelastography (TEG) and Rotational Thromboelastometry (ROTEM) have been increasingly used to diagnose fibrinogen deficiency and guide fibrinogen transfusion in trauma and surgical bleeding patients. We conducted a comprehensive and comparative review on the technologies and clinical applications of two typical functional fibrinogen assays using TEG (FF TEG) and ROTEM (FIBTEM) for assessment of fibrinogen level and deficiency, and prediction of transfusion requirement. Clot strength and firmness of FF TEG and ROTEM FIBTEM were the most used parameters, and their associations with fibrinogen levels as measured by Clauss method ranged from 0 to 0.9 for FF TEG and 0.27 to 0.94 for FIBTEM. A comparison of the interchangeability and clinical performance of the functional fibrinogen assays using the two systems showed that the results were correlated, but are not interchangeable between the two systems. It appears that ROTEM FIBTEM showed better associations with the Clauss method and more clinical use for monitoring fibrinogen deficiency and predicting transfusion requirements including fibrinogen replacement than FF TEG. TEG and ROTEM functional fibrinogen tests play important roles in the diagnosis of fibrinogen-related coagulopathy and guidance of transfusion requirements. Despite the fact that high-quality evidence is still needed, the two systems are likely to remain popular for the hemostatic management of bleeding patients.

## 1. Introduction

Fibrinogen is perhaps the most important protein in hemostasis, as the final stage of the coagulation cascade is converted to fibrin by thrombin and cross-linked by factor XIII. It also induces platelet activation and aggregation via binding to glycoprotein IIb/IIIa receptors on the surface of platelets, acting as the bridge for stable clot formation [1]. During major bleeding, fibrinogen is the first clotting factor to reach critically low levels below the normal physiological level of around 2 to 4 g/L, which is associated with increased bleeding, coagulopathy, and in turn worsened clinical outcomes [2–5]. Fibrinogen is an independent predictor of mortality in major trauma patients [6].

Thromboelastography (TEG; Haemonetics Corporation, Haemoscope Division, Nile, Illinois, USA) and Rotational Thromboelastometry (ROTEM; Tem Innovations GmbH, Munich, Germany; succeeded by Instrumentation Laboratory, Bedford, MA, USA) are two point-of-care systems for hemostatic tests in whole blood [7]. Both have been increasingly used to diagnose fibrinogen deficiency [8], predict risk of bleeding and mortality, and guide fibrinogen transfusion in trauma [9], cardiac surgery [10], liver transplantation [11], and postpartum bleeding [12]. TEG- and ROTEM-based algorithms have been widely used to direct fibrinogen administration in different settings leading to reduction in transfusion needs, costs, adverse outcomes, and even mortality [13–16] although a recent review indicated that the benefit of reduced blood products (red blood cells, fresh frozen plasma, and platelet) and improved morbidity in bleeding patients with the application of TEG- or ROTEM-guided transfusion strategies were primarily based on trials of elective cardiac surgery involving cardiopulmonary bypass, with low-quality evidence [17]. Similarly, a systematic review and meta-analysis of the use of TEG and ROTEM in goal-directing treatment with allogeneic blood products in bleeding patients found that the amount of transfused red blood cells, fresh frozen plasma, and bleeding volume was reduced in the TEG- or ROTEM-guided groups compared to the control groups either treated at the clinician's discretion or based on conventional coagulation tests (CCTs), whereas there were no differences in platelet transfusion and mortality [18]. Furthermore, a randomized clinical trial has concluded that TEG-guided massive transfusion protocol for severe trauma improved survival compared with that guided by CCTs (i.e., prothrombin time [PT]/international normalized ratio [INR], fibrinogen, and D-dimer) and utilized less plasma and platelet transfusion during the early phase of resuscitation [19].

Nevertheless, another recent review on routine use of viscoelastic blood tests for diagnosis and treatment of coagulopathic bleeding concluded that TEG and ROTEM tests only reduced red blood cells and platelet transfusion in adults undergoing cardiac surgery [20]. Meta-analyses across all indications (trauma, cardiac surgery, and liver transplantation) have shown that TEG and ROTEM tests are cost-saving and more effective than standard laboratory tests in trauma patients and patients undergoing cardiac surgery [21]. Moreover, studies, including ours, have shown that TEG and ROTEM provide different results for diagnosing coagulopathy and guiding transfusion [22–24]. Further evaluation of TEG- or ROTEM-guided transfusion in prospective case-matched studies and different patient categories with low risk of bias is needed. Alternatively, TEG and ROTM have also been used to study the effects of fibrinogen supplementation on hemostasis in an* in vitro *model of dilutional coagulopathy [25], the correction of dilutional coagulopathy by fibrinogen concentrate in orthopedic patients [26], and hypothermic coagulopathy [27] as well as in patient blood [28].

Although the role of TEG and ROTEM in diagnosis of coagulopathy, prediction of transfusion and mortality in bleeding patients has been reviewed [12, 29]. This paper is focused on the studies on TEG and ROTEM functional fibrinogen tests: FF TEG and ROTEM FIBTEM for the diagnosis of fibrinogen deficiency and prediction of transfusion requirements in bleeding patients. A follow-up paper will be focused on the use of FF TEG and ROTEM FIBTEM to guide fibrinogen replacement and monitor its hemostatic effects.

The paper is structured into four major sections. The first session describes the principles of the two systems and various commercially available tests with an emphasis on functional fibrinogen tests. The second session reviews the use of TEG and ROTEM for measurement of functional fibrinogen levels in relation to fibrinogen concentration assays. The third session discusses the diagnosis of fibrinogen related coagulopathy and prediction of transfusion requirements in different clinical settings. Finally, the review compares the two systems in terms of reagents, parameter values, and clinical uses.

## 2. Principles of TEG and ROTEM Functional Fibrinogen Tests

TEG 5000 (Haemonetics Corporation, Haemoscope Division, Niles, IL, USA) and ROTEM delta (Instrumentation Laboratory, Bedford, MA, USA) are the most used viscoelastic hemostasis analyzers. Both systems measure the viscoelastic properties of blood as it clots under low shear stress based on pin-and-cup technology, but there are primary hardware differences between the two [68]. The TEG analyzer has a pin suspended by a torsion wire, wherein a cup oscillates through 4.75 degree/5 seconds. In contrast, the ROTEM analyzer has an immobile cup, wherein a pin/wire transduction system oscillates through 4.75 degree/6 seconds. It has been suggested that the ROTEM system uses a ball-bearing system for power transduction, which makes it less susceptible to movement and vibration [69] and that the automatic pipetting may ensure less variations among operations [70].

In addition to the differences in instrument, the two viscoelastic point-of-care systems used different reagents for various tests and applications [68]. Specifically, the functional fibrinogen reagent for TEG is composed of lyophilized tissue factor and a platelet inhibitor (abciximab) that binds to glycoprotein IIb/IIIa receptors to inhibit platelet aggregation and exclude the platelet contribution to clot strength [71]. However, it could contain kaolin or celite instead of tissue factor [32, 43]. ROTEM fibrinogen assay uses two solutions called ex-tem and fib-tem [71]. The ex-tem solution contains a combination of recombinant tissue factor and phospholipids that activates the extrinsic pathway of the coagulation system, while the fib-tem solution contains CaCl_2_ as a recalcification reagent and a platelet inhibitor (cytochalasin D) that inhibits actin/myosin-system. A new reagent called fib-tem plus contains 2 platelet inhibitors, cytochalasin D, which inhibits platelet cytoskeletal reorganization, and tirofiban, which is a glycoprotein IIb/IIIa inhibitor similar to abciximab that prevents fibrinogen from binding to glycoprotein IIb/IIIa receptors on the surface of platelets and platelet aggregation [48]. Furthermore, single portion reagents composed of all lyophilized reagents required for each ROTEM test have been developed [72].

Although multiple parameters can be measured for blood coagulation and fibrinolysis, maximum amplitude MA in TEG and maximum clot strength MCF in ROTEM have been mostly used as a direct measure of fibrinogen functions. According to each manufacturer, the normal range of MA as measured by FF TEG using citrated blood is 11-24 mm (Guide to functional fibrinogen) and the normal range of MCF as measured by ROTEM FIBTEM assay is 7-24 mm (Instructions for use of fib-tem). In addition, the clot firmness at 5, 10, and 15 min after CT (A5, A10, and A15) has been reported.

It should be noted that new and fully automated (no pipetting) TEG and ROTEM systems (TEG 6s and ROTEM sigma) are now commercially available. Both work with 4-channel cartridges based on different mechanisms. TEG 6s uses a new technology called coagulation resonance analysis and works with two types of cartridges. One is a global hemostasis cartridge to perform four tests (kaolin TEG, kaolin TEG with heparinase, RapidTEG, and FF TEG) simultaneously and the other is a Platelet Mapping cartridge to perform the TEG Plate Mapping test (kaolin TEG, activator F (ActF), adenosine diphosphate (ADP), and arachidonic acid (AA) [73, 74]. ROTEM sigma operates on the proven pin and cup technology as ROTEM delta, but uses two types of cartridges containing lyophilized beads reagents instead of liquid reagents for four tests per cartridge (cartridge 1: FIBTEM, EXTEM, INTEM, APTEM; cartridge 2: FIBTEM, EXTEM, INTEM, HEPTEM) [75].

## 3. Methods

A PubMed search was completed for the TEG and ROTEM studies involving functional fibrinogen tests. The search terms were as follows: thrombelastogra*∗* or thromboelastogra*∗* or TEG and fibrinogen, thromboelastomentr*∗* or thrombelastromentr*∗* or ROTEM and fibrinogen. The specific clinical use/interest of TEG and ROTEM (e.g., trauma, cardiac surgery, transfusion, hypofibrinogenemia, hypo, and hyperfibrinolysis) was also used as keywords in the search. Abstracts were used to determine the relevance and when appropriate, further review of the original articles was warranted. Additional publications were selected from the cross-references listed in the original papers and from the citing articles, and additional search was made through Medline, Scopus, and Institute of Scientific Information databases for those topics with limited findings from PubMed. The search was primarily focused on human studies.

We analyzed the correlations between functional fibrinogen assays, i.e., Clauss method and FF TEG or ROTEM FIBTEM tests, and the accuracies (sensitivity, specificity, and area under receiver operating characteristic (ROC) curve) of FF TEG and ROTEM FIBTEM for the detection of hypofibrinogenemia and prediction of bleeding and transfusion. Furthermore, data were extracted and pooled from different trials where FF TEG MA, ROTEM FIBTEM MCF versus plasma fibrinogen concentration data were reported. Linear regression with 95% confidence interval was then performed using SigmaPlot Version 13.0 (Systat Software, Inc., San Jose, California, USA). We also calculated likelihood ratios of FF TEG and ROTEM FIBTEM for the diagnosis of hypofibrinogenemia defined as plasma fibrinogen level < 1.5 g/L using the following formula: sensitivity/(1-specificity) [76]. Meta-analysis of the clinical benefit of functional fibrinogen assays using TEG and ROTEM within their different uses could not be conducted due to the small sample size and heterogeneity of studies.

## 4. Measurement of Fibrinogen Levels and Functions

TEG has been used to study* in vitro* effects of fibrinogen on coagulation of plasma deficient in coagulation factors and diluted by colloids [77, 78], and the effect of a cardiopulmonary bypass system with biocompatible coating on fibrinogen levels [38]. ROTEM has been used to determine the usefulness of fibrinogen substitution to reverse dilutional coagulopathy in* in vitro* and* in vivo* models [79–81]. All these studies showed that, to various extents, fibrinogen improved clot strength (MA or MCF), clot formation (R or CT), and clot propagation (Alpha) as measured by TEG or ROTEM.

Clinical studies showed variable correlations between maximum clot strength/firmness and fibrinogen levels as summarized in Table 1. The correlation coefficients range from 0 (no significant correlation) [37] to 0.9 (strong correlation) [42] for FF TEG and 0.27 [3] to 0.94 [47] for ROTEM FIBTEM. The correlations between A5, A10, or A15 and fibrinogen levels have also been reported [34, 44, 45, 51, 53, 57, 59]. These early values of clot firmness can provide fast and reliable prediction of MCF to guide haemostatic therapy in severe bleeding [82]. Other ROTEM tests (e.g., EXTEM) showed certain degrees of correlations with plasma fibrinogen concentrations as well [50, 55]. Both FF TEG and ROTEM FITBEM have been used in clinical settings of trauma [30, 32–34, 46], cardiac surgery [36, 37, 39, 49, 52], liver transplantation [42, 54], and pregnancy [43, 59] with different popularities. In addition, ROTEM FIBTEM has been used for assessment of fibrinogen function in neurosurgery [60], burn injury [61], and cirrhosis [62]. The variations in the correlations could be due to the differences in study population and range of fibrinogen concentrations as most TEG and ROTEM tests were performed using TEG 5000 and ROTEM delta with the same reagent and procedures as recommended by their manufacturers. For example, one study reported no significant correlations between either TEG functional fibrinogen level (FLEV) or maximal amplitude (MA) and Clauss fibrinogen level at baseline or 10 min postprotamine in cardiac surgery patients [37]. The different correlations were also reported for children at different ages [40, 41].

**Figure 1:**
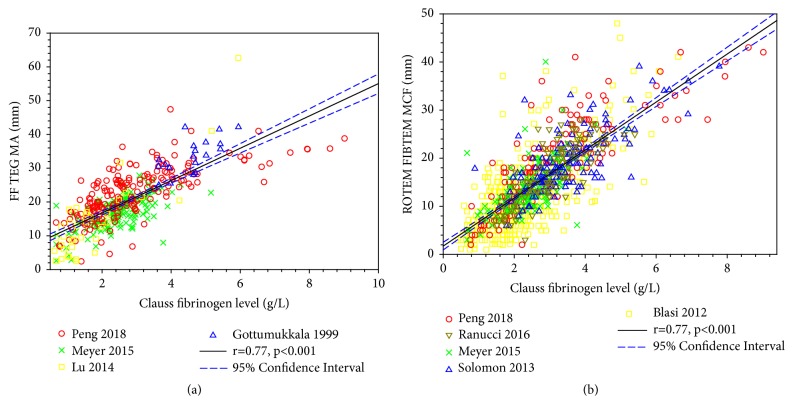
Correlations of Clauss fibrinogen level with FF TEG MA (a) and with ROTEM FIBTEM MCF (b). The correlation coefficients were obtained through linear regression of all data extracted from the literature. The data were pooled from different clinical studies involving a total of 275 patients for the correlation between Clauss fibrinogen level and FF TEG MA [30, 35, 42, 43] and a total of 626 patients for the correlation between Clauss fibrinogen level and ROTEM FIBTEM MCF [30, 35, 48, 52, 56]. The means ± standard deviations of FF TEG MA and Clauss fibrinogen level in (a) are 20.14±8.28 mm and 2.71±1.32 g/L. The means ± standard deviations of ROTEM FIBTEM MCF and Clauss fibrinogen level in (b) are 15.40±7.86 mm and 2.74±1.22 g/L.

Figure 1(a) shows the correlation between plasma fibrinogen concentration measured by the Clauss method and FF TEG MA based on the data extracted from several studies. Overall correlation from pooled literature data was strong. Similarly, Figure 1(b) shows an overall strong correlation between the Clauss fibrinogen and FIBTEM MCF from pooled data extracted from the literature.

The Clauss assay is considered a standard functional test for fibrinogen concentration by determination of the time in seconds to clot formation following addition of excess thrombin [83]. Other methods such as prothrombin time-derived method [56] and enzyme-linked immunosorbent assay (ELISA) [83] are also used. ELISA does not discriminate between functional and non-functional immunoreactive fibrinogen protein, and even some degraded forms of fibrinogen [84].

The Clauss method is limited to only small concentrations of heparin (which inactivates thrombin through anti-thrombin III), which is a serious limitation in cardiac surgery. It may be affected by fibrin degradation products, polymerization inhibitors as other inhibitors of fibrin formation [85]. Its turnaround time is approximately 40 min [86]. In comparison, TEG and ROTEM function fibrinogen assays can be completed in 15 min and can provide rapid and accurate detection of hyperfibrinolysis [87]. Another advantage of TEG and ROTEM is that they can be used for fully heparinised patients, e.g., when on cardiopulmonary bypass, with the use of a heparinase TEG cup or heparinase.* In vitro* studies showed that FF TEG MA was unaffected by heparin levels up to 2.8 IU/mL, but was reduced at 5.6 IU/mL of heparin in blood even performed in heparinase TEG cups [71], while ROTEM FIBTEM MCF was insensitive to heparin up to a concentration of 4 IU/ml in whole blood, but then declined to values less than 50% of baseline at 8 IU/mL [88]. A clinical study in pediatric cardiac surgery validated the use of FIBTEM in the presence of very high heparin concentrations (400 IU/kg bodyweight) [89].

The correlations between Clauss fibrinogen concentration and FF TEG MA or ROTEM FIBTEM MCF could be affected by a number of factors in addition to heparin concentration. As elucidated in Table 1, different reagents and instruments produced by Stago, Siemens, and Instrumentation Laboratory are used to quantify fibrinogen concentration. It is known that there can be systematic differences between the fibrinogen concentrations obtained with various commercial kits [90]. The different detections used in the Clauss method and resuscitation fluids administered may affect the correlation [91, 92]. This is likely due to the fact that FIBTEM test is more affected than fibrinogen concentration assays by the fluids [93]. Solomon et al. examined correlations between ROTEM FIBTEM MCF and Clauss fibrinogen concentration as determined using photo-optical, mechanical, and electromechanical detections in cardiac surgery patients [91]. The correlations obtained from the photo-optical and electromechanical methods (r=0.82 and 0.81) were greater than the mechanical method (r=0.73 and 0.71). Fenger-Eriksen et al. assessed fibrinogen levels in plasma diluted* in vitro* with different fluids (isotonic saline, hydroxyethyl starch, and human albumin) using an antigen determination, three photo-optical Clauss methods, one mechanical Clauss method, a prothrombin-derived method, and viscoelastic measurement through ROTEM [92]. The fibrinogen levels were overestimated using the photo-optical Clauss methods as a result of the dilution with hydroxyethyl starch, whereas ROTEM FIBTEM MCF was reduced by the dilution and to a lesser extent by human albumin. The former was ascribed to an unexplained interference with the optical source by hydroxyethyl starch and the latter was due to impairment of fibrin polymerization induced by the fluid. In addition, the fibrinogen measurement by the Clauss method for the same set of plasma samples can vary within and between laboratories [94].

On the other hand, it was found that plasma fibrinogen level (FLEV) estimated by FF TEG was on average 1.0 g/L higher than that determined by the Clauss method in both surgical patients and healthy controls [95]. This is consistent with other reports of higher TEG FLEV values than the Clauss values in cardiac surgery [39] and overestimation of plasma fibrinogen level in liver transplantation when the plasma fibrinogen level becomes less than 1 g/L [42].

In our subgroup analysis of trauma patients who received fibrinogen concentrate versus placebo (i.e., normal saline), the correlation coefficients are not as significantly altered between the two groups (0.68 versus 0.67 for FF TEG and 0.88 versus 0.82 for ROTEM FIBTEM). This is in contrast with patients undergoing liver transplantation [42, 54] and cardiac surgery [52, 91] where the correlation was impaired by severe hypofibrinogenemia and fibrinogen replacement. Specifically, the correlations between FIBTEM MCF and Clauss fibrinogen concentration decreased from r=0.71-0.82 to 0.33-0.59 after administration of fibrinogen concentrate in patients undergoing complex cardiovascular surgery [91]. In addition, hyperfibrinogenemia (>4 g/L) could impair the correlation between ROTEM FIBTEM MCF and fibrinogen levels as reported in major upper gastrointestinal surgery [96]. Therefore, the discrepancy may be due to the differences in the range of plasma fibrinogen concentrations among these studies (e.g., interquartile range of 1.88-3.63 g/L in our study vs. 0.77-1.38 g/L in the liver transplantation study). Similar correlations were reported between FIBTEM clot amplitude and fibrinogen concentration (r=0.86) at admission and then decreased correlations (r=0.43 and 0.63) after admission in the trauma patients receiving fibrinogen concentrate [44].

It should be noted that the concentration measurements by the Clauss method and other plasma fibrinogen assays cannot be the same as the clot strength of whole blood measured by TEG and ROTEM. Apparently, fibrinogen is not the only contributor to clot amplitude in these FF TEG and ROTEM FIBTEM assays, which may impose some limitations on FF TEG and ROTEM FIBTEM for the assessment of fibrinogen deficiency. Activated FXIII and hematocrit could have an impact on clot firmness as well and affect the correlations [49, 96–99]. Postoperative FXIII levels correlated to FIBTEM MCF more significantly than fibrinogen levels in patients undergoing major upper gastrointestinal surgery [96]. However, the same study also showed a significant correlation between platelet count and ROTEM FIBTEM MCF (r=0.55), which implied that the test might be profoundly impaired by incomplete inhibition of the platelet contribution to the clot strength. Furthermore, FXIII levels might affect FF TEG as well [98].

In addition, Ogawa et al. reported a higher correlation between ROTEM FIBTEM MCF and Clauss plasma fibrinogen at lower hematocrit (<25%) than at higher hematocrit (>30%) (r =0.88 and 0.67, resp.) in cardiac surgery [99]. In contrast, Solomon et al. found no significant differences between the lowest haematocrit group (<25%) and the higher haematocrit groups (25-27.9%, 28-29.9%, and >30%) for FIBTEM MCF or fibrinogen concentrations in whole blood and plasma, and thus the hematocrit effect appeared to be negligible [49]. FF TEG has shown hypocoagulable states in patients with cyanotic congenital heart disease mainly due to impaired fibrinogen function negatively affected by elevated haematocrit [100].

In addition to MA, other TEG parameters, e.g., estimated functional fibrinogen level (FLEV) and kinetic time K and Alpha, kaolin TEG K and Alpha, have shown different extents of correlations with fibrinogen concentration [32, 33, 47]. Kornblith et al. confirmed a significant correlation between FF TEG FLEV and the Clauss fibrinogen assay in trauma patients in agreement with the published findings from Harr et al., but the correlation as assessed by linear regression was weaker (R^2^= 0.57 vs. 0.87) [32, 33]. In addition, different correlations of FLEV with kaolin TEG MA (R^2^= 0.44-0.64 vs. 0.80), K (R^2^= 0.01 vs. 0.35), and Alpha (R^2^= 0.03 vs. 0.70) were reported in their studies likely due to different statistical methods (linear vs. polynomial regression). The correlations were affected by fibrinogen concentration, decreases at low and high ranges [32]. FF TEG FLEV was diminished and negatively correlated to haematocrit [100].

We observed moderate correlations of Clauss fibrinogen concentration with FF TEG K and Alpha (Spearman's correlation *ρ*=-0.46 and 0.40) and with ROTEM FIBTEM CFT and Alpha (*ρ*=-0.41 and 0.54) in trauma patients [30]. Furthermore, there were weak correlations of fibrinogen concentration with ROTEM FIBTEM CT (*ρ*=-0.29), and with FF TEG CL30 and ROTEM FIBTEM LI30 (*ρ*=0.21 and 0.20). The correlations between K/CFT, Alpha and fibrinogen concentration are consistent with their measurement of the activity of clotting factors, in particular fibrinogen [101], and are comparable with or stronger than the reported correlations between FF TEG K/Alpha and fibrinogen concentration [32, 33]. A linear correlation was observed between the clot shear elasticity G calculated from FF TEG MA and functional fibrinogen levels measured by the Clauss method in both whole blood (R^2^=0.605) and platelet-poor plasma (R^2^=0.94) [102].

Among all the parameters, the strongest correlations between FF TEG MA/ROTEM FIBTEM MCF and plasma fibrinogen concentration have been reported [32, 33, 103], suggesting these parameters are most useful for monitoring the role of fibrinogen in hemostasis of bleeding patients.

Together with kaolin TEG, FF TEG has been used to characterize functional fibrinogen to platelet ratio and was found useful in preoperatively identifying thrombotic complication in patients undergoing microvascular free tissue transfer in head and neck surgery [104]. FF TEG MA correlated with a number of biomarkers of endothelial activation and damages such as syndecan-1, thrombomodulin and protein C, and plasminogen activator inhibitor-1 (r=-0.37, p<0.001) in patients with severe sepsis [105].

## 5. Diagnosis of Coagulopathy/Hypofibrinogenemia and Prediction of Blood Transfusion

Table 2 summarizes the predictive accuracy of FF TEG and ROTEM FIBTEM in various clinical settings. MA and MCF are the main parameters used for the predictions of hypofibrinogenemia and blood transfusions. The prediction accuracy was evaluated by sensitivity, specificity, and area under the receiver operating characteristic curve (AUC) and variate regression analyses. Different cut-off values of fibrinogen concentrations ranging from 1 to 1.8 g/L were used to define hypofibrinogenemia. Traditionally, a plasma fibrinogen level of 1 g/L was established for fibrinogen replacement in patients with congenital fibrinogen deficiency, whereas the threshold varied from 0.8 to 2.0 g/L in patients with acquired fibrinogen deficiency [14]. In contrast, a critical fibrinogen concentration of 2.29 g/L was identified in trauma below which a significant increase in mortality occurred [106]. The discrepancy implies that the negative impact of fibrinogen deficiency in trauma may have been underestimated. It should also be noted that hypofibrinogenemia prevalence in major bleeding varies across clinical contexts [107].

Most clinical studies are prospective observational, while a few are retrospective and randomized controlled. Sample size ranged from 23 to 1077 patients. In contrast with ROTEM, FF TEG has been used less to detect hypofibrinogenemia and predict blood transfusion requirements with a focus on trauma patients. Among various clinical settings, ROTEM FIBTEM has been mostly used in trauma, cardiac surgery, and liver transplantation with best predictive power for hypofibrinogenemia (fibrinogen <1.5 g/L) (AUC=0.99) in cardiac surgery [47]. Furthermore, several studies have shown that FF TEG and ROTEM FIBTEM could predict bleeding and transfusion requirements in trauma [63, 64], cardiac surgery [52], and liver transplantation [58, 66] with various accuracies. It appeared that ROTEM would have better predictive accuracy than TEG because it has greater specificity for some common coagulopathies in cardiac surgery, such as fibrinogen deficiency. The averaged likelihood ratio of FF TEG MA for diagnosis of hypofibrinogenemia is 4.71±2.18 based on a number of studies [30, 35, 40], while the corresponding value of ROTEM FIBTEM MCF is 9.24±2.64 calculated from the literature [30, 35, 55].

Two studies evaluated ROTEM devices in patients with postpartum hemorrhage (PPH). One study provided data on the ability of ROTEM FIBTEM to predict hypofibrinogenemia (<1.5 g/L) [59]; the other evaluated the predictive power of ROTEM FIBTEM and Clauss fibrinogen for PPH and found no associations between the prepartum coagulation parameters and blood loss defined as blood loss ≥ 500 mL [67]. Alternatively, one study showed that FF TEG MA with a cut-off value of 12.1 mm could predict obstetric complications in nonpregnant dysfibrinogenemia patients with a sensitivity of 100%, specificity of 69.2%, and AUC of 0.923, but could not distinguish patients with bleeding and nonbleeding symptoms [108].

Only a few studies demonstrated ROTEM FIBTEM provided faster and better prediction than plasma fibrinogen concentration for massive transfusion [64] and bleeding [66], respectively. ROTEM FIBTEM provided early prediction of massive transfusion in trauma similar to the most predictive laboratory parameters (e.g., fibrinogen and hemoglobin concentrations) [64]. A separate study comparing standard fibrinogen measurement methods (i.e., Clauss method and thrombin clotting time) with ROTEM FIBTEM in patients with cirrhosis suggested FIBTEM as a promising alternative to standard plasma fibrinogen measurement in cirrhotic patients, especially in evaluating fibrin polymerization disorders in these patients [62].

There is insufficient evidence or low-quality evidence for the benefits of TEG and ROTEM for the prediction of bleeding and adverse outcomes beyond that achieved using routinely measured baseline factors or conventional coagulation tests (CCTs) except for rapidity. ROTEM EXTEM and FIBTEM were no better than routine laboratory tests for detecting differences between surviving and nonsurviving critically ill patients [109]. ROTEM FIBTEM was unable to predict PPH and not superior to CCTs in a prospective observational study of 217 healthy pregnant women [67]. On the other hand, ROTEM FIBTEM was not a good test to predict the presence of acute coagulopathy of trauma defined as an international normalized ratio (INR) > 1.3 or a fibrinogen level < 1.5 g/L unless combined with EXTEM, and either of the tests could predict the need for emergent blood product transfusions (defined as ≥5 units of red blood cells (RBC) and ≥3 units of plasma within the first 24 h of care) [65]. The use of CCTs such as INR in trauma has been severely criticized due to the lack of association with bleeding and blood transfusion. It has been reported that INR overestimated coagulopathy should not be used to guide blood transfusion in stable trauma and surgical patients [110].

Finally, if fibrinogen deficiency has a causal relationship with bleeding and adverse clinical outcomes, it is sensible to suggest that TEG and ROTEM functional fibrinogen tests that improve clinical prediction for fibrinogen-related bleeding may also have the potential to predict adverse clinical outcomes. However, randomized trials are needed to provide high-quality evidence for the role of TEG and ROTEM in diagnosis, management, and monitoring of fibrinogen function and replacement in bleeding patients.

It is noteworthy that recently two large randomized trials, CRASH-2 (Clinical Randomisation of an Antifibrinolytic in Significant Haemorrhage 2) [111] and WOMAN (World Maternal Antifibrinolytic) [112], provide high-quality evidence for the early use of tranexamic acid for a survival advantage to many bleeding patients. An* in vitro* study showed that the antifibrinolytic reduced fibrinogenolysis in addition to the correction of fibrinolytic effects on TEG (MA, LY) values in the presence of tissue plasminogen activator [113].

## 6. Comparison between TEG and ROTEM for Functional Fibrinogen Assays

A number of studies compared the reagents and devices between FF TEG and ROTEM FIBTEM. Solomon et al. showed that FF TEG MA was larger than FIBTEM MCF when performed with either their standard assay reagents (lyophilized tissue factor and abciximab on TEG and a combination of ex-tem and fib-tem on ROTEM) or the same assay reagent [71]. In addition, the FF TEG reagent produced higher values than the FIBTEM reagent on both TEG and ROTEM. Schlimp et al. compared different fibrinogen assays in eliminating platelet effects on TEG MA and ROTEM MCF. It was found that abciximab based on glycoprotein IIb/IIIa inhibition was less effective at inhibiting the platelet contribution to clot strength than cytochalasin D based on prevention of platelet cytoskeletal reorganization, resulting in larger FF TEG MA compared to ROTEM FIBTEM MCF and affecting their correlations and changes with fibrinogen concentration. In addition, the combination of both inhibitions provided the most accurate assessment of the clot strength and fibrinogen function [114]. It has been speculated that the ROTEM FIBTEM reagent might contain stabilizing agents (e.g., dimethyl sulfoxide) and more tissue factors than the FF TEG reagent [115]. These results are consistent with other studies comparing TEG and ROTEM functional fibrinogen assays for whole blood from surgical patients [48, 116], trauma patients, and healthy volunteers [91, 114].

However, the differences between FF TEG MA and ROTEM FIBTEM MCF obtained using the same reagents [71, 115] imply that TEG system itself may also be a contributing factor. The hardware differences between the two systems include the mechanisms for cup/pin rotation, detection of the rotation, cup materials, and interior surface properties [117, 118].

Meyer et al. compared different TEG and ROTEM tests including FF TEG and FIBTEM, and Clauss method for detection of trauma-induced coagulopathy and goal-directed transfusion therapy [34]. Specifically, FF TEG and ROTEM FIBTEM early amplitudes (A5, 10) and MA/MCF had similar correlations with Clauss fibrinogen level and could differentiate coagulopathic and transfused patients from noncoagulopathic and nontransfused patients.

Prüller et al. compared fibrinogen assays using FF TEG and ROTEM FIBTEM in surgical patients in terms of their MA and MCF values, and correlations with Clauss fibrinogen level [116]. It was found that FF TEG MA was higher than ROTEM FIBTEM MCF and their MA and MCF corresponded to different Clauss fibrinogen levels. The FF TEG MA showed a weaker correlation with Clauss fibrinogen than ROTEM FIBTEM MCF (R^2^=0.542 vs. 0.671).

Different TEG (Rapid, Kaolin, and FF) and ROTEM tests (EXTEM, INTEM, and FIBTEM) were compared for their sensitivity to detect fibrinolysis induced by tissue plasminogen activator in whole blood [119]. Compared to other tests, FF TEG and ROTEM FIBREM provided more rapid detection of fibrinolysis, but FF TEG detected changes in clot strength as well. Comparison of tissue factor-triggered ROTEM FIBTEM and EXTEM with contact-activated kaolin TEG in patients undergoing liver transplantation showed the highest and lowest hyperfibrinolysis detection rates by FIBTEM and kaolin TEG, respectively, suggesting the effects of coagulation activators and platelet inhibitors on sensitivity to identifying hyperfibrinolysis [120].

In contrast with hyperfibrinolysis detection, we found only kaolin TEG and ROTEM EXTEM as the methods of measuring hypofibrinolysis (also called fibrinolysis shutdown) instead of FF TEG and ROTEM FIBTEM. In one study of 914 trauma patients (ISS≥15), the threshold for hypofibrinolysis was determined for EXTEM ML at 5.5% with a sensitivity of 61.6% and specificity of 58.4% [121]. The study reported 29.9% hypofibrinolysis. In another study of 550 severe trauma patients with a median ISS of 19, EXTEM ML<3.5% was selected to define hypofibrinolysis with a sensitivity 42.5% and specificity 76.5% [122]. The method identified 25.6% hypofibrinolysis. A number of studies have used TEG in particular kaolin TEG LY30<0.81% to detect hypofibrinolysis in trauma patients with a median ISS of 25 [123, 124]. These studies reported fibrinolysis phenotypes with different prevalence: hypofibrinolysis at 29.9%, 25.6%, and 46%; physiologic fibrinolysis at 63.0%, 70.7%, and 36%. The ROTEM method indicated physiologic fibrinolysis as the predominant phenotype with 63% [121] and 71% [122] followed by hypofibrinolysis, while the TEG method showed hypofibrinolysis as the most common phenotype with 46% followed by physiologic fibrinolysis with 36% [124]. The discrepancy could be the difference in patients' characteristics (e.g., ISS) and the method itself (tissue factor-activated ROTEM EXTEM vs. contact-activated kaolin TEG). A recent retrospective analysis of the Pragmatic, Randomized Optimal Platelet and Plasma Ratios (PROPPR) trial found 61% of hypofibrinolytic patients as determined by kaolin TEG LY30<0.9%, followed by 22% of hyperfibrinolytic patients based on LY30≥3% [125]. The study also suggested that hypofibrinolysis did not reflect shutdown of enzymatic fibrinolysis with hypercoagulability, but rather a type of coagulopathy characterized by fibrinolytic activation with concurrent fibrinogen consumption and platelet dysfunction.

There is a lack of studies to compare the utilities of TEG and ROTEM for diagnosis of coagulopathy, prediction of mortality, and the requirement for massive transfusion, although both have been reported useful [45, 126]. We conducted a comparative study of functional fibrinogen assays using TEG and ROTEM in trauma patients [30] to determine (1) their interchangeability of the key parameter values obtained by the two systems in all trauma patients as well as severe trauma patients randomized to receive their fibrinogen concentrate or placebo (normal saline), and (2) utility of each system for predicting clinical outcomes and monitoring any changes in coagulation profiles in the trauma patients randomized for treatment with fibrinogen concentrate or placebo. In addition, a crossover analysis (ex-tem and fib-tem on TEG) was conducted to confirm whether the assay reagents or the device could contribute to the observed differences. Overall, we found that TEG and ROTEM parameter values were correlated, being strongest between MA and MCF, but were significantly different, and their agreement fell outside acceptable limits and thus their values were not interchangeable, arguably due to differences in both devices and assay reagents used. Specifically, ROTEM FIBTEM MCF had a higher correlation with Clauss fibrinogen (*ρ*=0.87 vs. 0.75) and lower value than FF TEG MA (17.1±8.0 mm vs. 22.4±7.5 mm). Clinically, TEG MA and ROTEM MCF showed reasonable predictive accuracy for plasma transfusion, but poor accuracy for any red blood cells and cryoprecipitate transfusions. Both well predicted hypofibrinogenemia (fibrinogen concentration < 1 g/L) with AUC of 0.95 and 0.96. ROTEM FIBTEM MCF seems to be more consistent with the duration of the between-group difference as indicated by fibrinogen levels than FF TEG MA. In addition, ROTEM FIBTEM detected changes in coagulation time (CT) and clot lysis (LI30) over hospitalization time as a result of fibrinogen treatment.

**Figure 2:**
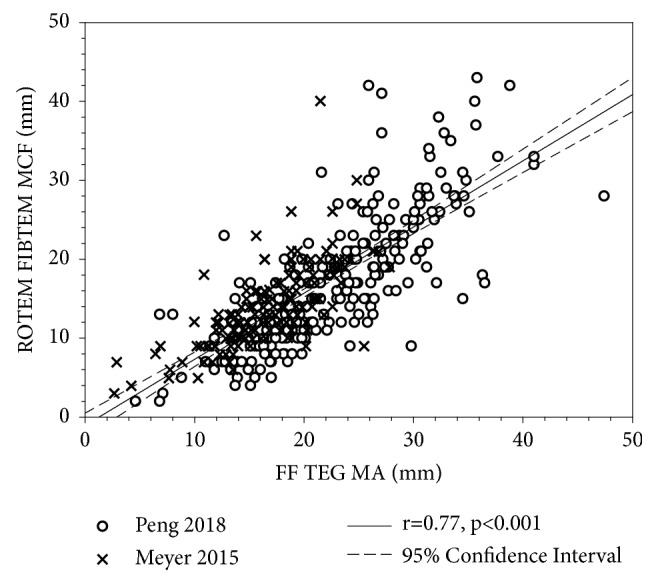
Comparison of FF TEG MA values and ROTEM FIBTEM MCF values. The correlation coefficient was obtained through linear regression of pooled data from Peng 2018 [30] and Meyer 2015 [35]. MCF=0.8384MA-1.065 (R^2^=0.5938). The means ± standard deviations of FF TEG MA and ROTEM FIBTEM MCF are 20.63±7.11 mm and 16.23±7.73 mm (n=363).

In a study similar to ours, TEG and ROTEM were compared for functional fibrinogen assays in trauma to determine specific cut-offs of TEG MA and ROTEM MCF for an increased risk of receiving a transfusion [35]. It was found that FF TEG MA and ROTEM FIBTEM MCF correlated well (*ρ*=0.71, p<0.001) and had the same correlation coefficient with Clauss fibrinogen level (*ρ*=0.64, p<0.001).  Figure 2 shows a strong correlation between FF TEG MA and ROTEM FIBTEM MCF (r=0.77, p<0.001) based on the pooled data of our study [30] and Meyer et al. [35], but a larger FF TEG MA than ROTEM FIBTEM MCF on average (20.63±7.11 mm vs. 16.23±7.73 mm, n=363, p<0.001).

## 7. Conclusions

TEG and ROTEM functional fibrinogen tests play important roles in diagnosis of fibrinogen-related coagulopathy and transfusion requirements including fibrinogen replacement. The clot strength of FF TEG and ROTEM FIBTEM is the most used parameter for discrimination of fibrinogen deficiencies and their correlations with Clauss fibrinogen levels are varied depending on patient population and range of fibrinogen concentrations. When using FF TEG and ROTEM FIBTEM to diagnose fibrinogen deficiency and predict transfusion requirements other variables such as hematocrits, factor XIII levels and fibrinogen concentrate ranges should be taken into consideration. Both TEG and ROTEM have been used to detect systemic fibrinolysis (physiologic, hypo, and hyperfibrinolysis). Studies comparing FF TEG and ROTEM FIBTEM suggest a stronger correlation of the latter with plasma fibrinogen concentration, likely due to its more effective elimination of platelet contribution to clot strength. We should be aware that the studies supporting the use TEG and ROTEM are limited for trauma and surgical bleeding patients. Even without robust clinical data, TEG and ROTEM are likely to remain popular for the hemostatic management of bleeding patients.

## Figures and Tables

**Table 1:** Summary of correlations between functional fibrinogen (FF) TEG/ROTEM FIBTEM and fibrinogen levels.

Clinical settings	Study population and blood sample	TEG and ROTEM methods	Results	Ref.
FF TEG				

Trauma	A randomized controlled trial of trauma patients at risk of significant hemorrhage (n=45, ISS=18-29) receiving either 6 g fibrinogen concentrate (RiaSTAP™) or placebo (normal saline). Citrated whole blood was collected from the randomized trauma patients at admission, 1-, 3-, 11-, 23- and 47-h post-infusion time.	Standard FF TEG was performed on a computerized TEG Hemostasis System 5000 (Haemonetics Corporation, Haemoscope Division, Niles, IL, USA) according to manufacturer's protocol. Specifically, 500 *μ*L of the blood sample was pipetted into a FF vial which contains lyophilized tissue factor with platelet inhibitor (abciximab) and gently mixed by inversion five times, and then 340 *μ*L of the mixture from the FF vial was added into a TEG cup pre-warmed to 37°C containing 20 *μ*L of 0.2 M calcium chloride. Plasma fibrinogen levels were measured by the standard von Clauss method.	FF TEG MA strongly correlated with Clauss fibrinogen concentration determined by Spearman's correlation (*ρ*=0.75, p<0.001). FF TEG K, Alpha showed moderate correlations with fibrinogen concentration (*ρ*=-0.46 and 0.40, p<0.001), while TEG FF CL30 only showed week correlations with fibrinogen concentration (*ρ*=0.21, p=0.004).	[30, 31]
	A prospective observational study of trauma patients (n = 68), with a median ISS of 23.5. Citrated whole-blood samples were obtained from the patients on arrival to the emergency department and within the first 5 days of admission to the surgical intensive care unit.	The FF and kaolin TEG assays were performed on the TEG 5000 device in the trauma research laboratory. The FF TEG assay measures the FF level (FLEV), which is extrapolated from the MA fibrinogen value. Plasma fibrinogen levels were measured by the standard von Clauss method.	Significant correlations between TEG FLEV and Clauss fibrinogen levels (R^2^ = 0.87, p<0.0001) and between TEG FF MA and Clauss fibrinogen levels (R^2^ = 0.75, p<0.0001). Moderate inverse correlation between FLEV and K (R^2^ = 0.35, p<0.0001), between FLEV and alpha (R^2^ = 0.70, p<0.0001).	[32]
	A prospective observational study of 251 critically injured trauma patients with a median ISS of 9 (1-19) at a single Level 1 trauma center. Citrated whole blood was collected from the patients on arrival and at 2, 3, 4, 6, 12, 24, 48, 72, 96, and 120 h after admission.	For the kaolin TEG, 340 *μ*L kaolin-activated blood was transferred to the TEG cup, pre-warmed to 37°C and containing 20 *μ*L of 0.2 M CaCl_2_. For the FF TEG, 500 *μ*L of citrated blood was added to the FF vial (kaolin + glycoprotein IIb/IIIa antagonist) and mixed; 340 *μ*L was then transferred to the TEG cup. Plasma fibrinogen concentration was assayed by the von Clauss method.	FLEV calculated by analytical software through a transformation of the FF MA to approximate the concentration of functional fibrinogen correlates with standard Clauss fibrinogen (R^2^= 0.57, p<0.001), similar to MA (R^2^=0.44-0.64, p<0.001) better than the kaolin TEG measures of fibrinogen function (kinetic time and angle) (R^2^=0.01, p=0.095; and R^2^=0.03, p=0.004)	[33]
	A prospective observational study of 182 trauma patients with a median ISS of 17 (9-26). Citrated blood was sampled immediately upon arrival.	The FF TEG with tissue factor activator and a platelet inhibitor (ReoPro, a GPIIb/IIIa inhibitor) was performed by a TEG 5000 Hemostasis Analyzer System using TEG Analytical Software version 4.2.3 (Haemonetics Corp., Braintree, MA), according to the manufacturer's recommendations.	FF TEG MA, A5, A10 had moderate correlation with fibrinogen concentration determined by Spearman's correlation (*ρ*=0.64, 0.68, 0.68, p<0.01).	[34, 35]

Cardiac surgery	A prospective observational study of 117 patients operated for ischemic heart disease. Blood was collected before cardiopulmonary bypass	FF TEG test was conducted using functional fibrinogen reagent (lyophilized tissue factor with platelet inhibitor- glycoprotein-IIb/IIIa receptor blocker). Analytical software calculated the functional fibrinogen level (FLEV) through the transformation of the MA value. Fibrinogen levels were assessed by the von Clauss method.	A moderate correlation was found between fibrinogen level and FF TEG FLEV with a Spearman correlation coefficient of 0.476 (p<0.0001).	[36]
	A prospective study of 160 cardiac surgery patients. Blood was collected at baseline, prior to heparinisation and 10 min post protamine administration	TEG-functional fibrinogen test was conducted with native blood using the functional fibrinogen reagent (lyophilized tissue factor with platelet inhibitor- glycoprotein IIb/IIIa receptor blocker). Analytical software calculates the functional fibrinogen level (FLEV) through the transformation of the MA value. Fibrinogen levels were assessed with citrated blood by the von Clauss method using Fibrinogen-CXL (HemosiL; Instrumentation Laboratory, Bedford, MA, USA) performed on the ACL TOP 300 CTS analyser (Instrumentation Laboratory, Bedford, MA, USA).	No significant correlation between the TEG FLEV and the Clauss fibrinogen level at the baseline (R^2^=0.106) and 10 min post protamine (R^2^=0.025) and between the TEG FF MA and the Clauss fibrinogen concentration at the baseline (R^2^=0.061) and 10 min post protamine (R^2^=0.26)	[37]
	A prospective study of 60 elective patients operated for ischemic heart disease with CPB and randomly assigned to a group with a heparin-coated CPB system or a group with a conventional (non-coated) circuit. Blood was collected from right after induction of general anesthesia, 2-h post operation, and second postoperative day.	FF TEG test was conducted by modified TEG with platelet inhibition (Haemoscope Corporation, Niles, IL, USA). Plasma fibrinogen levels were determined by a Clauss method not specified.	Spearman's correlation analysis showed a moderately positive correlation between perioperative Clauss fibrinogen level and FF TEG MA (n=60, r=0.408, p=0.002)	[38]
	A prospective non-randomized study of 51 cardiac surgery patients. Citrated (3.2%) blood collection tubes were used for all samples 3 time points: (1) baseline; (2) rewarming on cardiopulmonary bypass; (3) post bypass.	FLEV assays were determined by TEG 5000 hemostasis analyzers in accordance with company's protocol utilizing the provided functional fibrinogen reagent vials (heparinase cups were used for all bypass samples). Plasma fibrinogen levels were measured using the Clauss method (TriniCLOT Fibrinogen Kit with Destiny Max Coagulation Analyzer; Tcoag, Bray, Ireland).	A simple linear regression model showed a strong correlation between the standard laboratory assay (Clauss) and the functional fibrinogen level (FLEV) assay (r = 0.76; p<0.0001) of the fibrinogen values at the baseline. Similar correlation was seen at the rewarming and post bypass. FLEV values were consistently higher than the standard laboratory assay.	[39]
	A prospective observational study of 105 children less than 5 years of age undergoing congenital heart surgery with CPB. Citrated blood was collected before and after bypass.	FF TEG was performed on the TEG 5000 hemostasis analyzers with functional fibrinogen reagent vial and heparinase TEG cup (Haemonetics, Niles, IL) by a single technician, within 20 min of collection of the samples. Fibrinogen levels were determined by the Clauss method on platelet-poor, centrifuged blood samples (STA Fibrinogen, Diagnostica Stago).	Linear correlation coefficients between FF TEG MA and fibrinogen levels were 0.36 (p<0.001) before and 0.52 after bypass (p=0.02).	[40]
	A retrospective and observational study of 119 children younger than 10 years old undergoing congenital cardiac surgery with CPB. Blood was collected twice during surgery, after anesthesia induction and CPB.	ROTEM FIBTEM was performed according to manufacturer's procedure. Fibrinogen concentration was measured by a fully automated device (Diagnostica Stago, Asnières, France).	Post-CPB fibrinogen levels were not correlated with post-CPB FIBTEM MCF in infants (r=0.155, p=0.197, 0.155), whereas they were correlated with FIBTEM MCF in children older than 12 months (r=0.311, p=0.031).	[41]

Liver transplantation (LT)	A prospective study of 27 consecutive adult LT patients. Blood sample was taken from an arterial line at the time of the skin incision (the baseline) and 30 min after graft reperfusion	The whole blood was analyzed with TEG 5000 hemostasis analyzer according to the manufacturer's instructions. Plasma fibrinogen level were measured with a modified Clauss method using a coagulation analyzer (STA-R Evolution Expert series hemostasis system, Diagnostica Stago, Parsippany, NJ, USA)	FF TEG MA correlated strongly with the plasma fibrinogen level at the baseline (Spearman's correlation coefficient *ρ*=0.90, p<0.01); however, the correlation reduced after the graft reperfusion (*ρ*=0.58, p<0.01). The same correlations were seen between FF TEG FLEV and the plasma fibrinogen level.	[42]

Pregnancy	A prospective study of 21 healthy, term parturients scheduled for elective cesarean delivery. Fresh whole blood was drawn from each patient.	Modified TEG was performed with 360 mL of 1% celite-activated whole blood and with 5 mL of (2mg/mL) ReoPro® (platelet aggregation inhibitor) added to 355 mL of 1% celite-activated whole blood within 4 min of blood collection. The plasma fibrinogen concentration was measured by the Clauss quantitative fibrinogen assay using thrombin derived from bovine plasma (Ortho Diagnostic System Inc., Raritan, NJ).	Linear regression analysis revealed TEG MA as a significant predictor of the plasma fibrinogen level, with an adjusted R^2^ of 0.49, and a slope of fibrinogen level= 9.56×MA + 150.68	[43]

ROTEM FIBTEM				

Trauma	Randomized controlled trial of trauma patients at risk of significant hemorrhage (n=45, ISS=18-29) receiving either 6 g fibrinogen concentrate (RiaSTAP™) or placebo (normal saline). Citrated whole blood was collected from the randomized trauma patients at admission, 1-, 3-, 11-, 23- and 47-h post-infusion time.	Standard ROTEM FIBTEM was performed on a ROTEM delta system according to manufacturer's protocol. Specifically, analyses were performed using 300 *μ*L of citrated whole blood and 20 *μ*L of ex-tem together with 20 *μ*L of fib-tem following the procedure as recommended by the company. Plasma fibrinogen levels were measured by the standard von Clauss method.	ROTEM FIBTEM MCF strongly correlated with Clauss fibrinogen concentration determined by Spearman's correlation (*ρ*=0.87, p<0.001). ROTEM FIBTEM CFT, Alpha showed moderate correlations with fibrinogen concentration (*ρ*=-0.41 and 0.54, p<0.001), while CT and LI30 weekly correlated with fibrinogen concentration (*ρ*=-0.29, p<0.001 and 0.20, p=0.003).	[30, 31]
	A prospective study of 517 adult trauma patients with a systolic blood pressure (SBP) of < 90 mmHg and a median ISS of 14 (8–27). Citrated blood was collected within 20 min of arrival in the emergency department (ED).	Blood samples were analyzed within 2 h of blood draw, with a ROTEM delta instrument, at 37°C. Two separate ROTEM assays were performed for each patient, the EXTEM, measuring tissue factor-initiated clotting, and the FIBTEM, with the addition of cytochalasin D, a platelet inhibitor as per manufacturer's protocols. Fibrinogen levels were determined with the Clauss method using STA Fibrinogen (Stago, Asnières sur Seine, France) and Siemens Thrombin (Sysmex UK, Milton Keynes, UK) reagents.	EXTEM and FIBTEM measures of A5 and maximal clot formation (MCF) were significantly correlated with Clauss fibrinogen levels, and the correlations between FIBTEM A5 and MCF were slightly stronger than EXTEM (r^2^ = 0.44 vs. 0.35 and 0.27 vs. 0.26). EXTEM and FIBTEM A5 gave a receiver operating characteristic curve area of 0.8 (95% confidence interval 0.7–0.9, p<0.001) for discriminating patients with admission fibrinogen levels below 1.5 g/L.	[3]
	A retrospective study of 358 trauma patients with a median ISS of 26 (17–34). Citrated blood was collected at admission and during the first 12-h care.	EXTEM and FIBTEM were performed in a standardized fashion within 30 min of blood collection. Fibrinogen concentration was measured by the Clauss technique, STA-Fibrinogen.	Correlations between fibrinogen concentration and FIBTEM A5 at admission (Spearman coefficient *ρ*=0.858) and during care (*ρ*=0.824), no blood product group (*ρ*=0.772) and blood product group (*ρ*=0.823).	[44]
	A prospective observational cohort study of 182 trauma patients with a median ISS of 17 (9 to 26). Blood was sampled immediately upon arrival at hospital and kept at room temperature.	EXTEM, INTEM and FIBTEM assays were performed with citrated blood according to the manufacturer's recommendations 1 h after sampling.	Fibrinogen concentration had moderate correlations with A5, A10 and MCF of EXTEM (*ρ*=0.65-0.68), INTEM (*ρ*=0.62-0.68) and FIBTEM (*ρ*=0.68).	[34]
	A prospective observational study of 88 trauma patients with an ISS of 22 (12-34). Blood samples were collected immediately after the patient's arrival to the trauma room and at 6, 12 and 24 h after admission	EXTEM, INTEM and FIBTEM were performed at 37°C in parallel with the citrated blood within 2 h and after 15 min of collection in a standardized way. Fibrinogen levels were assayed according to Clauss technique using Fibriquick® reagent (Biomérieux).	A significant correlation was found between fibrinogen levels and EXTEM CT (r = 0.40, p<0.001), A15 (r=0.69, p<0.001), between fibrinogen levels and INTEM A15 (r=0.66, p<0.001), and between fibrinogen levels and FIBTEM A10 (r=0.85, p<0.001)	[45]
	A prospective cohort study of 334 blunt trauma patients (ISS≥15 or Glasgow Coma Score ≤14). Citrated blood was collected at hospital admission.	ROTEM EXTEM and FIBTEM tests were performed according to manufacturer's guides. Plasma fibrinogen concentration was measured using test kits from Siemens Healthcare AG, Erlangen, Germany.	EXTEM and FIBTEM MCF showed strong correlations with fibrinogen concentration (*ρ*=0.79 and 0.81, respectively, p<0.001).	[46]

Cardiac surgery	A prospective, observational pilot study of 35 patients undergoing elective cardiac surgery on cardiopulmonary bypass for cyanotic congenital heart disease. Blood samples were collected after induction of anesthesia.	EXTEM, INTEM and FIBTEM were performed with citrated blood after recalcification with 20 *μ*L CaCl_2_. Fibrinogen concentration assay was not provided.	Fibrinogen concentration showed a significant correlation with ROTEM FIBTEM MCF (Pearson coefficient r=0.94, p< 0.001), but not with EXTEM MCF (r=0.077, p=0.67) and INTEM MCF (r=0.162, p=0.37).	[47]
	A prospective observational study of 30 patients undergoing cardiac surgery with cardiopulmonary bypass (CPB). Citrated blood was drawn at the beginning of surgery (pre-CPB), 20 minutes before weaning from CPB and 5 minutes after heparin neutralization.	TEG with the functional fibrinogen reagent (FF TEG), ROTEM with fib-tem (FIBTEM) and fib-tem plus containing two platelet inhibitors: cytochalasin D and tirofiban (FIBTEM PLUS) were run for a minimum of 30 min. Fibrinogen concentration was measured using the Clauss method and photo-optical determination on the ACL Top 700 (Instrumentation Laboratory, Milan, Italy) and QFA thrombin reagent (Instrumentation Laboratory).	Significant positive correlations were found between MCF or MA and fibrinogen concentration (all p<0.001); the highest correlation was with FIBTEM PLUS MCF (Spearman coefficient *ρ*=0.70), followed by FIBTEM (*ρ*=0.66) and FF TEG (*ρ*=0.56).	[48]
	A prospective study of 157 patients undergoing cardiac surgery with CPB. Citrated blood were collected at baseline (before induction of anaesthesia) and at the end of CPB (after protamine administration).	Whole blood FIBTEM was performed using a ROTEM device according to the manufacturer's instructions at each time point. Plasma fibrinogen concentration was measured using the Clauss method and whole blood fibrinogen concentration was calculated as plasma fibrinogen concentration × (100 − haematocrit)/100.	The Spearman correlation coefficient between FIBTEM MCF and plasma fibrinogen concentration was 0.68 at baseline and 0.70 after protamine, while that between FIBTEM MCF and whole blood fibrinogen concentration was 0.74 at baseline and 0.72 after protamine (all p<0.001).	[49]
	A prospective observational study of 35 patients undergoing elective cardiac surgery with CPB. Citrated blood was collected from at three different time points: preoperatively (immediately before anesthesia induction), and at 1- and 24-h post operation.	Kaolin TEG and ROTEM EXTEM, INTEM, FIBTEM were conducted with the citrated blood within 1 h after the collection, according to the manufacturer's instructions. Fibrinogen concentration was measured by a standard method (not specified).	Correlations between fibrinogen concentration and EXTEM MCF (Pearson coefficient r=0.71, p<0.0005), INTEM MCF (r=0.53, p=0.001), FIBTEM MCF (r=0.79, p<0.0005) were found at 1-h post operation and correlations between fibrinogen concentration and TEG K (r=-0.52, p=0.002), Alpha (r=0.53, p=0.001), TEG MA (r=0.63, p<0.0005), EXTEM MCF (r=0.58, p=0.001), INTEM MCF (r=0.63, p<0.0005), FIBTEM (r=0.50, p=0.003) at 24-h post operation.	[50]
	A retrospective observational study of 1077 patients undergoing cardiac surgery with CPB. Citrated blood was collected during the rewarming phase (≥36°C).	EXTEM and FIBTEM were conducted at 37°C as per manufacturer's reagents and procedures. Fibrinogen concentration was measured by the Clauss method using STAR Evolution (Stago, Paris, France).	Clauss fibrinogen concentration was correlated strongly with EXTEM MCF and A10 (Spearman coefficient *ρ*=0.68 and 0.70; p<0.01) and FIBTEM MCF and A10 (*ρ*=0.78 and 0.78; p<0.01). The correlation was related inversely to hemoglobin concentration (p< 0.01).	[51]
	A randomized controlled trial of 116 high-risk patients undergoing cardiac surgery with CPB. Blood was collected at 20 min before removal of the aortic cross-clamp (baseline) and after placebo or fibrinogen administration.	FIBTEM test was conducted. Fibrinogen concentrations were measured according to a photo-optical Clauss method, with a coagulation analyser (ACL TOP 700), a calibrator (Hemosil Normal Control), and a thrombin reagent (Hemosil QFA thrombin).	Linear regression analyses showed a good association between FIBTEM MCF and Clauss fibrinogen concentration at the baseline population (R^2^=0.66, p=0.001), which reduced to R^2^=0.16 (p=0.003) in fibrinogen-supplemented subjects.	[52]
	A prospective observational study of 110 patients undergoing cardiac surgery with CPB. Citrated whole blood was sampled from a central venous line or from the extracorporeal circuit at pre-CPB, on-CPB, post-CPB.	ROTEM assays of INTEM, EXTEM, FIBTEM and HEPTEM were performed at 37°C by certified bioanalytical technicians. Plasma levels of fibrinogen were measured using the Clauss technique on a coagulation analyzer (BCS, Dade Behring Inc., Germany) using the Multifibren U-Reagent according to manufacturer's specifications.	The fibrinogen level and FIBTEM A10 were significantly correlated for all data points (Pearson coefficient r=0.81; p<0.05). Their correlation was stronger on-CPB at a mean hemoglobin of 83 g/L (r= 0.87) and post-CPB (mean hemoglobin 88 g/L; r=0.74) than pre-CPB (mean Hemoglobin 105 g/L; r=0.66).	[53]

Liver transplantation (LT)	A retrospective observational study of 282 patients receiving liver transplantation. Citrated blood was collected at 1 h after induction of general anesthesia, 1 h after the first surgical incision, 30 min after hepatectomy, 30 min after graft reperfusion, and after hepatic artery anastomosis.	ROTEM tests (EXTEM, INTEM and FIBTEM) were routinely performed according to the manufacturer's instructions. Fibrinogen concentration was measured using the Dade thrombin reagent (Siemens Healthcare Diagnostics, Erlangen, Germany) and an automatic coagulation analyzer (Sysmex CA-7000, Siemens Healthcare Diagnostics, Erlangen, Germany).	Fibrinogen was the primary determinant of FIBTEM MCF, accounting for 73% of the variability. However, in severe hypofibrinogenemia (fibrinogen <1 g/L), fibrinogen accounted only 22% of FIBTEM MCF variability. Spearman's correlations between fibrinogen concentration and EXTEM MCF (*ρ*=0.66, p<0.001), INTEM MCF (*ρ*=0.65, p<0.001), FIBTEM MCF (*ρ*=0.83, p<0.001).	[54]
	A retrospective observational study of 295 patients (254 living donors and 41 LT patients). Citrated blood was collected from 1 h after induction of general anesthesia, 1 h after surgical incision, 30 min after hepatectomy, 30 min after graft reperfusion, and after hepatic artery anastomosis.	The same as above	FIBTEM MCF significantly correlated with fibrinogen concentration with a highest Spearman's coefficient (*ρ* = 0.84, p<0.001), followed by EXTEM MCF (*ρ*=0.67, p<0.001), INTEM MCF (*ρ*=0.66, p<0.001).	[55]
	A prospective study of 253 patients receiving orthotopic liver transplantation. Citrated blood samples were collected after induction of general anesthesia, at the end of the hepatectomy, 20 minutes after graft revascularization, and 90 minutes after graft revascularization.	The blood samples were tested just after collection by ROTEM gamma device operated according to manufacturer instructions and with the type and concentration of reagents as provided by Pentapharm (Munich, Germany). Fibrinogen concentration was measured by prothrombin time-derived method, with values below 2 g/L being checked by the Clauss method on a coagulometer (KC-1A, Amelung, Lemgo, Germany) using fibrinogen reagent (Diagnostica Stago, Asnières, France) according to the manufacturer's instructions.	FIBTEM MCF correlated with fibrinogen level (Spearman's correlation coefficient *ρ*=0.70).	[56]
	A prospective observational study of 20 patients undergoing orthotopic liver transplantation. Blood samples were taken at the following points during OLT: the beginning of the dissection phase, the end of dissection phase, 10 min into the anhepatic phase, 10 min into the reperfusion phase, and 1 h after donor graft reperfusion.	FIBTEM was performed with citrated blood within 4 h of collection according to the manufacturer's instructions. Clauss fibrinogen assay was performed on a CA-1500 analyzer (Sysmex, Milton Keynes, UK).	There was a significant correlation between FIBTEM MCF and Clauss fibrinogen concentration (Pearson's correlation coefficient r =0.75, p≤0.01).	[23]
	A prospective observational study of 23 patients undergoing orthotopic liver transplantation. Blood samples were collected after induction of general anaesthesia, during hepatectomy, at the anhepatic stage, 30–60 min after graft revascularization, at the end of surgery, and 24 h after surgery	ROTEM tests (EXTEM, INTEM, FIBTEM and APTEM) were performed in the operating theatre and by the anaesthesiologists treating the patients according to the manufacturer's instructions using equipment and test reagents provided by Pentapharm GmbH. Plasma fibrinogen concentration was determined by the Clauss method performed on ACL Top automates (Instrumentation Laboratory, Lexington, MA, USA)	ROTEM FIBTEM A10 correlated with Clauss fibrinogen (r=0.74, p<0.0001), only slightly stronger than EXTEM A10 (r=0.72, p<0.0001)	[57]

	A retrospective study of 401 patients who underwent liver transplantation. Blood was sampled at 1 h after induction of general anaesthesia, 1 h after surgical incision, 30 min after hepatectomy, and 30 min after graft reperfusion and after hepatic artery anastomosis.	ROTEM tests were performed according to the manufacturer's instructions, using equipment and test reagents provided by Tem International GmbH. Fibrinogen level was measured using the Dade Thrombin Reagent (Siemens Healthcare Diagnostics) and an automatic coagulation analyser (Sysmex CA-7000, Siemens Healthcare Diagnostics).	The correlations of FIBTEM A5, A10, MCF with fibrinogen levels were determined using Spearman's rank correlation coefficients (*ρ*= 0.75, 0.76 and 0.75, respectively, p<0.001).	[58]

Postpartum	A prospective observational study of 91 women at the third trimester of pregnancy: 37 with postpartum haemorrhage (study group) and 54 without abnormal bleeding (control group). Citrated blood was collected from women at the third trimester of pregnancy.	Standard FIBTEM was carried out by clinicians with the blood samples in the delivery room. Fibrinogen assay was for plasma concentration within 5 min after sampling with a STAR automated coagulation analyser (Diagnostica Stago Inc., Franconville, France)	A5, A15 and MCF were significantly lower in the haemorrhage group than in control (p<0.0001) and strongly correlated with fibrinogen levels both groups (Spearman's correlation coefficient *ρ*= 0.84–0.87, p<0.0001).	[59]

Neurosurgery	A prospective observational study of 92 patients undergoing emergent neurosurgery	ROTEM analyses were performed within min of blood sampling by anesthesia nurses or physicians trained to perform the ROTEM tests according to the manufacturer's instructions. Plasma fibrinogen concentration was determined by Clauss method (Siemens-Dade Behring Healthcare Diagnostics, Marburg, Germany).	There was a strong correlation between PTT and INTEM CT (r=0.76) as well as between fibrinogen concentrations and FIBTEM MCF (r =0.70).	[60]

Burn injury	A prospective observational study of 20 consecutive patients. Citrated blood was collected immediately after admission, 12, 24 and 48 h after admission.	Four ROTEM tests (INTEM, EXTEM, FIBTEM and APTEM) were performed simultaneously on the four-channel ROTEM in the ICU ward lab at each time point. Plasma fibrinogen level was determined by Clauss method using an automated coagulation analyzer (STA-R Evolution, Stago, Asnières, France).	Fibrinogen level and FIBTEM MCF were within the reference range until 24 h after burn injury, but increased significantly after 48 h. There was a significant correlation between FIBTEM MCF and fibrinogen level (Spearman's correlation coefficient *ρ* = 0.714, p <0.001).	[61]

Cirrhosis	A cross-sectional single-centre study involving 60 patients with alcoholic cirrhosis, 24 patients with cholestatic cirrhosis and a control group of 50 healthy volunteers. Blood samples were taken at admission.	ROTEM FIBTEM assay was performed with citrated blood within 1 h after collection using a set of standard reagents according to the manufacturer's recommendations. Clauss fibrinogen assay was performed using a BCS® XP automated coagulation analyzer (Siemens Healthcare Diagnostics GmbH, Marburg, Germany).	In all cirrhosis patients, FIBTEM MCF strongly correlated with fibrinogen concentration (r=0.72-0.77, p< 0.001).	[62]

CPB=Cardiopulmonary Bypass; ISS=Injury Severity Score; Liver transplantation=LT

**Table 2:** Clinical evaluation of TEG and ROTEM functional fibrinogen tests for diagnosis of coagulopathy (hypofibrinogenemia), prediction of transfusion requirements and mortality.

Clinical settings	Study design and patients	Blood collection and analysis	Findings	Ref.
FF TEG				

Trauma	Randomized controlled trial of trauma patients at risk of significant hemorrhage (n=45, ISS=18-29) receiving either 6 g fibrinogen concentrate (RiaSTAP™) or placebo (normal saline)	Citrated whole blood was collected from the randomized trauma patients at admission, 1-, 3-, 11-, 23- and 47-h post-infusion time. Standard FF TEG was performed on a computerized TEG Hemostasis System 5000 (Haemonetics Corporation, Haemoscope Division, Niles, IL, USA) according to the manufacturer's protocol.	FF TEG MA predicted hypofibrinogenemia (fibrinogen concentration < 1 g/L) and 24-h plasma transfusion with high accuracies (AUC=0.95, p=0.002 and AUC=0.70, p=0.042).	[30, 31]
	A prospective study of 182 adult trauma patients with a median ISS of 17 (9-26)	Blood was sampled immediately upon arrival to trauma centre and evaluated in tissue factor-activated and platelet inhibited TEG (i.e. FF TEG) precisely 1 h after sampling by a hemostasis analyzer system (TEG 5000, Haemonetics Corp., Braintree, MA) according to the manufacturer's recommendations. All analyses were conducted at 37°C.	Sensitivity, specificity and AUC of FF TEG MA for detection of fibrinogen < 1.5 g/L were 77%, 81% and 0.869, respectively. FF TEG MA was also a univariate predictors of massive transfusion (>10 units of RBCs) at 6 and 24 h with odd ratios of 0.79, 0.82 and mortality at 28 days with a hazard ratio of 0.84.	[35, 63]

Cardiac surgery	A prospective observational study of 105 children less than 5 years of age undergoing congenital heart surgery with CPB	Whole blood samples were collected via indwelling arterial catheters before and after CPB. FF TEG and kaolin heparinase TEG were performed on the TEG 5000 with company's reagents by a single technician, within 20 min of collection of the samples. Plasma fibrinogen levels were determined by the Clauss method using the commercial reagents and instrument (STA Fibrinogen, Diagnostica Stago).	FF TEG MA predicted hypofibrinogenemia (fibrinogen concentration < 2 g/L) with AUC of 0.71 (95% CI 0.59-0.83)	[40]

ROTEM FIBTEM				

Trauma	Randomized controlled trial of trauma patients at risk of significant hemorrhage (n=45, ISS=18-29) receiving either 6 g fibrinogen concentrate (RiaSTAP™) or placebo (normal saline)	Citrated whole blood was collected from the trauma patients at admission, 1-, 3-, 11-, 23- and 47-h post-infusion time. Standard ROTEM FIBTEM was performed on a ROTEM delta system (Tem Innovations GmbH, Munich, Germany; succeeded by Instrumentation Laboratory, Bedford, MA, USA) according to the manufacturer's protocol.	ROTEM FIBTEM MCF predicted hypofibrinogenemia (fibrinogen concentration < 1 g/L) and 24-h plasma transfusion with high accuracies (AUC=0.96, p<0.001) and AUC=0.72, p=0.023).	[30, 31]
	A prospective observational study of 88 trauma patients an median ISS score of 22 (12-34)	Blood samples were collected immediately after the patient's arrival to the trauma room (H0) and at 6 h (H6), 12 h (H12) and 24 h (H24) after admission, representing a total of 270 samples. The ROTEM measurements and standard coagulation tests were performed within 2 h of collection of blood samples.	Sensitivity, specificity and AUC of FIBTEM A10 for detection of fibrinogen < 1 g/L were 91%, 85% and 0.96, respectively.	[45]
	A retrospective analysis of data from 323 patients with an injury severity score (ISS) ≥16 (20-50)	Blood samples were taken immediately upon admission to ER. ROTEM analyses (EXTEM, INTEM, FIBTEM) were typically performed at the bedside within minutes of sample collection. Fibrinogen concentration was measured by the Clauss method (STA-Fib® assay (Roche Diagnostics GmbH); optical read-out), using a STA-Compact® machine (Roche Diagnostics GmbH, Vienna, Austria).	Sensitivity, specificity and AUC of FIBTEM A10/MCF for prediction of massive transfusion (≥10 units RBC transfused in 24 h) 63.3/77.5%, 83.2/74.9%, 0.83/0.84 (95% CI 0.78-0.87/0.79-0.88), similar to fibrinogen concentration	[64]
	A prospective cohort study of 517 trauma patients with a median ISS of 14 (8-27)	Blood was drawn from either the femoral vein or antecubital fossa into a 2.7-mL citrated vacutainer within 20 min of arrival in the emergency department (ED). ROTEM tests were performed within 2 h of blood draw with a ROTEM delta instrument, at 37°C.	Sensitivity, specificity and AUC of FIBTEM A5 for detection of fibrinogen <1.5 g/L 87%, 70% and 0.8 (95% CI 0.7-0.9)	[3]
	A prospective, single-center, non-interventional, non-controlled, open clinical study of 50 trauma patients with a median ISS of 13 (4-66)	Blood was collected at hospital admission, 3- and 24-h after admission and analyzed by ROTEM assays (EXTEM and FIBTEM). EXTEM was considered positive if one of the four principle parameters (CT, CFT, MCF, and Maximum Lysis) greater than 20% of the expected highest or lowest normal value of the manufacturer normal value ranges (CT ≥ 94, CFT ≥ 190, MCF ≤ 40, ML ≤ 12). FIBTEM was considered positive if MCF was at least 20% smaller than the expected mean normal value (MCF ≤ 7).	Sensitivity, specificity and AUC of FIBTEM MCF < 7 mm within normal EXTEM patients are 100%, 90.2%, 0.951 and 0%, 87.5%, 0.563 for predictions of coagulopathy (INR≥1.3) and mortality at 30 days	[65]
	A prospective study of 182 adult trauma patients with a median ISS of 17 (9-26)	Blood was sampled immediately on hospital arrival. FIBTEM assays were performed with citrated blood precisely 1 h after sampling according to the manufacturer's recommendations. Fibrinogen level was determined by Clauss method.	Sensitivity, specificity and AUC of FIBTEM MCF < 10 mm were 80%, 89% and 0.889 for detection of fibrinogen <1.5 g/L.	[35]

	A prospective cohort study of 334 blunt trauma patients (ISS≥15 or Glasgow Coma Score ≤14).	Citrated blood was collected at hospital admission. ROTEM tests were performed according to the manufacturer's instructions, using equipment and test reagents provided by Tem International GmbH. Logistic regression models were used to evaluate ROTEM tests for prediction of 24-h death and 6-h transfusions.	FIBTEM MCF with a cut-off of 7 mm predicted the need for RBC transfusion with an odd ratio of 0.92 (95% CI 0.87–0.98)	[46]

Cardiac surgery	A prospective, observational pilot study of 35 patients undergoing elective cardiac surgery on cardiopulmonary bypass (CPB) for cyanotic congenital heart disease	Citrated blood was collected after induction of anesthesia and analyzed by ROTEM. No details were provided. Fibrinogen concentration assay was not provided.	ROTEM FIBTEM MCF is highly predictive of hypofibrinogenemia (fibrinogen <1.5 g/L) (AUC=0.99).	[47]
	A randomized, placebo-controlled trail of 116 high-risk patients undergoing cardiac surgery with CPB	ROTEM FIBTEM was performed 20 min before removal of the aortic cross-clamp, after fibrinogen supplementation. Fibrinogen concentrations were measured upon arrival in ICU according to a photo-optical Clauss method.	FIBTEM MCF with the best cut-off value of 14 mm yielded a good discriminative power for severe bleeding with an AUC of 0.721, sensitivity of 80%, specificity of 72%	[52]
	A retrospective observational study of 1077 patients undergoing cardiac surgery with CPB.	Citrated blood was collected during the rewarming phase (≥36°C). EXTEM and FIBTEM were conducted at 37°C as per manufacturer's reagents and procedures. Fibrinogen concentration was measured by the Clauss method using STAR Evolution (Stago, Paris, France).	The optimal FIBTEM A10 cut-off for diagnosis of a fibrinogen concentration <1.5 g/L was ≤8 mm with an AUC of 0.95.	[51]
	A prospective observational study of 110 patients undergoing cardiac surgery with CPB.	Citrated whole blood was sampled from a central venous line or from the extracorporeal circuit at pre-CPB, on-CPB, post-CPB. ROTEM assays of INTEM, EXTEM, FIBTEM and HEPTEM were performed at 37°C by certified bioanalytical technicians. Plasma levels of fibrinogen were measured using the Clauss technique on a coagulation analyzer (BCS, Dade Behring Inc., Germany) using the Multifibren U-Reagent according to manufacturer's specifications.	An on-CPB FIBTEM A10 ≤ 10 mm identified patients with a post-CPB Clauss fibrinogen of ≤1.5 g/L with a sensitivity of 0.99 and a positive predictive value of 0.60.	[53]
	A retrospective and observational study of 119 children <10 years old undergoing congenital cardiac surgery with CPB.	Blood was collected twice during surgery, after anesthesia induction and CPB. ROTEM EXTEM and FIBTEM were performed with citrated blood according to the manufacturer's recommendations. Intraoperative excessive blood loss was defined as estimated blood loss ≥50% of estimated blood volume. Logistic regression models were used to identify predictors for excessive blood loss.	Post-CPB FIBTEM CA10<5 mm predicted massive blood loss with an odd ratio of 11.1 (95% CI 2.6-47.3, p=0.001) and AUC of 0.83.	[41]

Liver transplantation (LT)	A retrospective observational study of 295 patients (254 living donors and 41 LT patients).	Citrated blood was collected from 1 h after induction of general anesthesia, 1 h after surgical incision, 30 min after hepatectomy, 30 min after graft reperfusion, and after hepatic artery anastomosis. ROTEM® tests (EXTEM, INTEM and FIBTEM) were routinely performed according to the manufacturer's instructions. Fibrinogen concentration was measured using the Dade thrombin reagent (Siemens Healthcare Diagnostics, Erlangen, Germany) and an automatic coagulation analyzer (Sysmex CA-7000, Siemens Healthcare Diagnostics, Erlangen, Germany).	FIBTEM MCF < 8mm predicted hypofibrinogenemia (fibrinogen < 1.28 g/L) with a sensitivity of 82%, a specificity of 90% and AUC of 0.94.	[55]
	A prospective study of 253 patients receiving orthotopic liver transplantation.	Citrated blood samples were collected after induction of general anesthesia, at the end of the hepatectomy, 20 min after graft revascularization, and 90 min after graft revascularization. The blood samples were tested just after collection by ROTEM gamma device operated according to manufacturer instructions and with the type and concentration of reagents as provided by Pentapharm (Munich, Germany). Fibrinogen concentration was measured by the PT-derived method, with values below 2 g/L being checked by the Clauss method.	Sensitivity, specificity and AUC of FIBTEM A10 for detection of plasma fibrinogen level (<1.3 g/L) 86%, 55% and 0.801	[56]
	A retrospective, single-centre, observational study of 243 adult liver transplant patients	Blood samples were collected immediately upon admission to ICU and once daily until the seventh postoperative day. ROTEM tests including EXTEM, INTEM, and FIBTEM were performed. Standard laboratory tests (PT, aPTT, fibrinogen) were performed using a BCS Analyzer (Siemens Healthcare Diagnostics Products GmbH, Erlangen, Germany).	FIBTEM A10/MCF predicted postoperative bleeding with a sensitivity of 90/90%, specificity of 33/32%, AUC of 0.636/0.632, better than fibrinogen concentration with 74%, 39% and 0.531	[66]
	A prospective observational study of 23 patients undergoing orthotopic liver transplantation.	Blood samples were collected after induction of general anaesthesia, during hepatectomy, at the anhepatic stage, 30–60 min after graft revascularization, at the end of surgery, and 24 h after surgery. ROTEM tests (EXTEM, INTEM, FIBTEM and APTEM) were performed in the operating theatre and by the anaesthesiologists treating the patients according to the manufacturer's instructions using equipment and test reagents provided by Pentapharm GmbH. Plasma fibrinogen concentration was determined by the Clauss method performed on ACL Top automates (Instrumentation Laboratory, Lexington, MA, USA).	ROTEM FIBTEM A10 ≤8 mm predicted hypofibrinogenemia (fibrinogen < 1 g/L) with a sensitivity of 0.83, specificity of 0.35, and AUC of 0.61, worse than EXTEM with corresponding values of 0.83, 0.75 and 0.84	[57]

	A retrospective of 401 patients who underwent liver transplantation.	Blood was sampled at 1 h after induction of general anaesthesia, 1 h after surgical incision, 30 min after hepatectomy, and 30 min after graft reperfusion and after hepatic artery anastomosis. A total of 1125 FIBTEM tests were performed according to the manufacturer's instructions. Fibrinogen level was measured using the Dade Thrombin Reagent (Siemens Healthcare Diagnostics) and an automatic coagulation analyser (Sysmex CA-7000, Siemens Healthcare Diagnostics).	ROC curve analysis showed that a cut-off value of FIBTEM A5 at 4 mm and A10 at 5 mm predicted fibrinogen < 1 g/L with a sensitivity of 81% and 76%, specificity of 77% and 82%, AUC of 0.86 and 0.87	[58]

Postpartum hemorrhage	A prospective observational study of 91 women at the third trimester of pregnancy: 37 with postpartum haemorrhage (study group) and 54 without abnormal bleeding.	Standard FIBTEM was carried out by clinicians with citrated blood samples in the delivery room. Plasma fibrinogen was assayed within 5 min after sampling with a STAR automated coagulation analyser (Diagnostica Stago Inc., Franconville, France) according to standard procedures.	A cut-off value of A5 and A15 at 6 mm provided an sensitivity of 100% for both parameters, a specificity of 85 and 88%, and AUC of 0.96 and 0.97, respectively to detect a fibrinogen level <1.5 g/L in postpartum haemorrhage.	[59]
	A prospective observational pilot study including 217 healthy pregnant women	Blood samples were collected upon admission to the delivery room for labor and within 1 h after vaginal delivery. All ROTEM tests were performed with the recommended reagents and in accordance with the manufacturer's procedures. Fibrinogen levels were measured with STA-fibrinogen reagent (Roche Diagnostics GmbH, Mannheim, Germany) using Clauss method.	The AUC of ROTEM FIBTEM MCF for prediction of postpartum hemorrhage defined as blood loss ≥ 500 mL was 0.52 (95% confidence interval 0.41–0.64, p =0.699), similar to the predictive power of fibrinogen levels (AUC=0.53, 95% confidence interval 0.40–0.65, p=0.644).	[67]

Neurosurgery	A prospective observational study of 92 patients undergoing emergent neurosurgery	Blood was sampled in the operating theater on citrated tubes and ROTEM analyses were performed within min of blood sampling by anesthesia nurses or physicians trained to perform the ROTEM tests according to the manufacturer's instructions. Plasma fibrinogen concentration was determined by Clauss method (Siemens-Dade Behring Healthcare Diagnostics, Marburg, Germany).	The need for transfusion (≥ 3 PRBCs) was best predicted by EXTEM and FIBTEM MCF (AUC of 0.72 and 0.71, respectively) and by fibrinogen concentration (AUC of 0.70), with a sensitivity of 38.2, 33.3, 25.6% and specificity of 85.1, 96.2 and 100%.	[60]

AUC=Area under the receiver operating characteristic curve; CI=Confidence Interval; CPB=Cardiopulmonary Bypass; ICU=Intensive Care Unit; ISS=Injury Severity Score
